# A complex microsatellite at chromosome 7q33 as a new prognostic marker of colorectal cancer

**DOI:** 10.18632/oncotarget.21077

**Published:** 2017-09-16

**Authors:** Xu Ye, Hongyu Deng, Min Su, Qianjin Liao, Dan Huang, Duan-Fang Liao, Zhi-Qiang Xiao, Deliang Cao

**Affiliations:** ^1^ Research Center of Carcinogenesis and Targeted Therapy, Xiangya Hospital, Central South University, Changsha, Hunan 410008, China; ^2^ The Higher Educational Key Laboratory For Cancer Proteomics and Translational Medicine of Hunan province, Xiangya Hospital, Central South University, Changsha, Hunan 410005, China; ^3^ Hunan Cancer Hospital and The Affiliated Cancer Hospital of Xiangya School of Medicine, Central South University, Changsha, Hunan 410013, China; ^4^ Division of Stem Cell Regulation and Application, State Key Laboratory of Chinese Medicine Powder and Medicine Innovation in Hunan (incubation), Hunan University of Chinese Medicine, Changsha, Hunan 410208, China

**Keywords:** colorectal cancer, microsatellite, polymorphisms, survival, prognostic marker

## Abstract

Disease-specific markers are critical for early diagnosis, targeted therapy and prognostic prediction of diseases. Current study reports a complex microsatellite as a new prognostic marker of sporadic colorectal cancer. This microsatellite located at Chromosome 7q33 is composed of three tetranucleotide tandem repeats, (TTCC)_2_(TCCC)_5_(TCCT)_7_, flanked by a CT-rich sequence. We analyzed polymorphisms of this microsatellite in 158 sporadic colorectal cancer, 143 matched normal adjacent tissues (NAT) and 150 health donors. Our results showed that this complex microsatellite was instable with polymorphic frequency of 77.2% in colorectal cancer, 52.4% in NAT and 54.7% in health donors (p<0.01) when compared to reference sequence. In the three tandem repeats, (TCCT)_7_ site was most polymorphic accounting for over 70.0% of polymorphisms in this complex microsatellite, followed by (TTCC)_2_ site for approximately 20%. Polymorphisms in (TCCC)_5_ was rare. Polymorphisms at the (TCCT)_7_ site were mainly insertions of 1 to 4 copies of TCCT (88.6%), and deletions occurred in about 6.4% of cases. The (TTCC)_2_ site was featured with one copy TTCC insertions. Pair-wise analyses between colorectal tumors and NAT revealed that 88 of 121 (72.7%) tumors displayed expansion, contraction or both in these tetranucleotide tandem repeats when compared to NAT. A cross-analysis with clinicopathological data of 158 colorectal cancers revealed that polymorphic alterations of the microsatellite associated with less lymphatic metastasis (p<0.001), and the colorectal cancer patients with polymorphic changes in this microsatellite demonstrated better survival (n=112, p=0.0058). Together these data suggest that this complex microsatellite is a potential prognostic marker of sporadic colorectal cancer.

## INTRODUCTION

Colorectal cancer (CRC) is a leading cause of cancer-related deaths in the United States; approximately 1 in 19 Americans (5.29%) will suffer from colorectal cancer during their life-time [1]. Individuals who have inflammatory bowel disease (IBD), i.e., ulcerative colitis and Crohn’s disease, have high risk of developing colorectal cancer, which increases exponentially with duration of the disease; surveillance of colorectal cancer in IBD patients has important clinical impact in prevention of IBD-associated cancer death [2, 3].

Advanced colorectal cancer has a high mortality rate; and early diagnosis is the only practical way to improve the disease-free survival of cancer patients [4–6]. Nearly 75% of colorectal cancer develops among people free of IBD and a familial history of hereditary non-polyposis colorectal cancer (HNPCC), colorectal neoplasia, or familial adenomatous polyposis (FAP), so called sporadic colorectal cancer. This makes the early diagnosis difficult due to lack of screening [7–10]. Up to date, colonoscopy screening in populations of IBD, FAP and HNPCC or subjects over 50 years old is an effective procedure for the surveillance and early diagnosis of colorectal cancer and has reduced the mortality in past decades [11–13]. However, colonoscopy is invasive and uncomfortable with potential complications, such as postpolypectomy, postbiopsy bleeding, puncturing of the colon lining and postpolypectomy syndrome [14–16]. Colonoscopy is also costly and requires sedation and thorough preparation of the colon, which often results in reduced patient compliance. Fecal occult blood test (FOBT) is non-invasive and home-based and newer methods, such as Hemoccult SENSA and fecal immunochemical tests (FIT), have improved sensitivity; however, their specificity is limited, often leading to false diagnosis [17–19]. In addition, FOBT fails to detect non-bleeding polyps and has poor specificity for colorectal cancer and adenoma [17, 20]. Hence, it is needed to identify and validate non-invasive, affordable, highly sensitive and specific biomarkers for screening and early diagnosis and/or prognostic prediction of colorectal cancer to improve the clinical management of this lethal disease.

Microsatellites have been shown to be potent genetic markers of cancers. Microsatellite instability (MSI) often correlates with certain age-related diseases, including colorectal cancer [21–24]. Microsatellites are defined as 1 to 6 repetitive DNA sequences distributed throughout the genome [25, 26]. MSI is a form of genomic instability that results from molecular defects in DNA mismatch repair machinery, causing frameshift mutations in highly repetitive microsatellite sequences [21, 23, 27]. In hereditary non-polyposis colon cancer (HNPCC), a disease caused by the mutations of DNA mismatch repair genes *hMSH2* or *hMLH1*, MSI occurs in up to 90% of cases, compared to approximately 15% in sporadic tumors [21, 28]. A high level of MSI in colorectal cancer usually indicates a favorable response to adjuvant chemotherapy and better prognosis [25, 29].

This study found that a microsatellite located at Chromosome 7q33 functions as a prognostic marker of colorectal cancer. It is a complex microsatellite composed of three tetranucleotide tandem repeats, (TTCC)_2_(TCCC)_5_(TCCT)_7_, flanked by CT-rich sequences. Our results demonstrated that this complex microsatellite is instable and that its polymorphic alterations were associated with less lymph node metastasis of colorectal cancer and better patient survival.

## RESULTS

### Identification of a complex repetitive sequence at Chromosome 7q33

**Figure 1:**
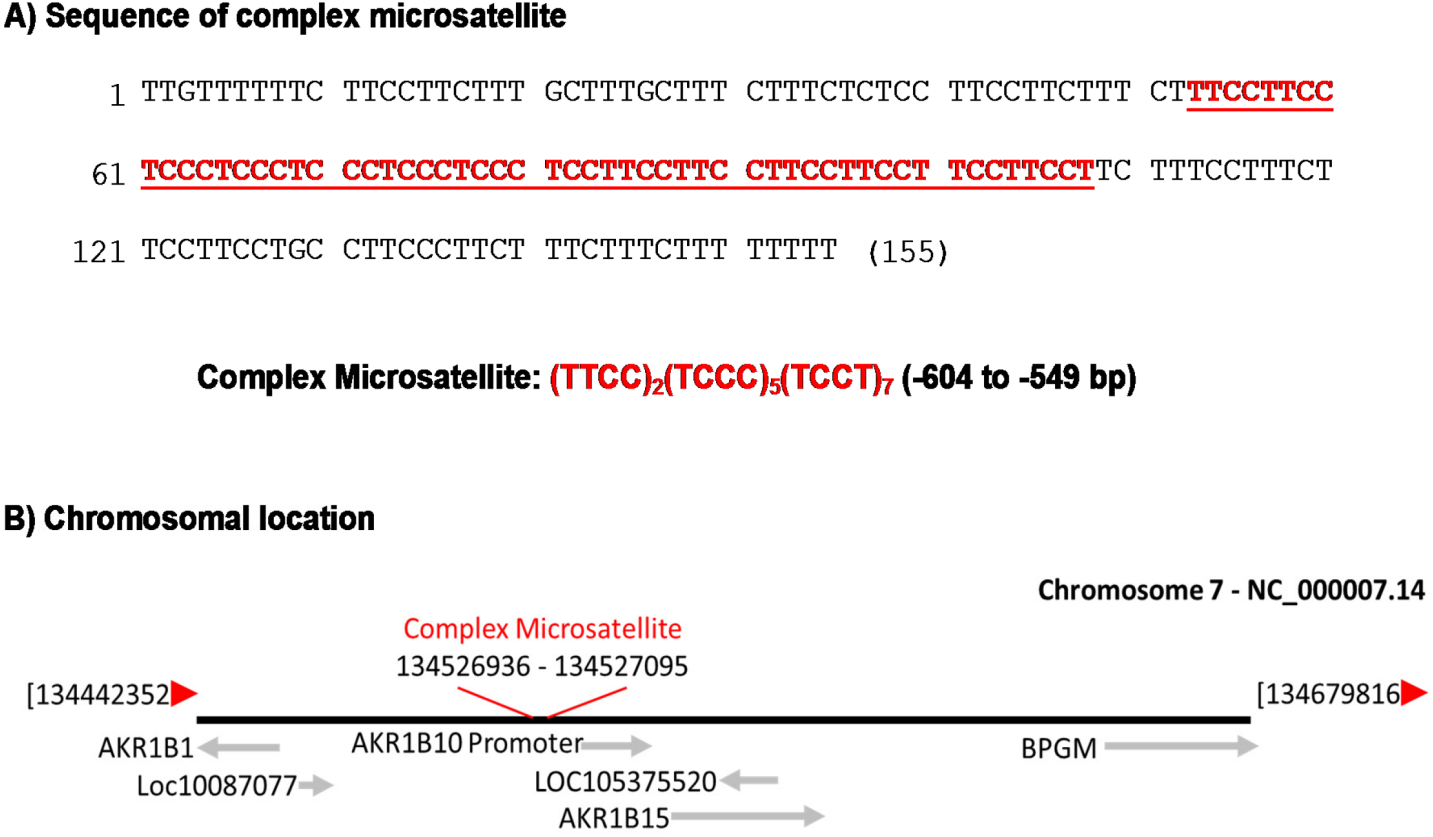
A complex microsatellite at Chromosome 7q33 **(A)** Sequence and components of this microsatellite, consisting of three consecutive tetranucleotide tandem repeats flanked by a C and T- enriched sequence. **(B)** Diagram of the chromosomal location of this microsatellite.

DNA sequence analysis of the AKR1B10 promoter region, a gene frequently silenced in inflammatory bowel disease and colorectal cancer [31, 32], revealed a repetitive sequence composed of tandem 4-nucleotide repeats, (TTCC)_2_(TCCC)_5_(TCCT)_7_, with a flanking C and T- enriched sequence (Figure 1A). This repetitive sequence is located at Chromosome 7 q33 (NC_000007.14, nucleotide number 134526936 - 134527095) (Figure 1B). In this study, we used this sequence as a reference sequence.

### Polymorphisms of this complex microsatellite in colorectal cancer

**Figure 2:**
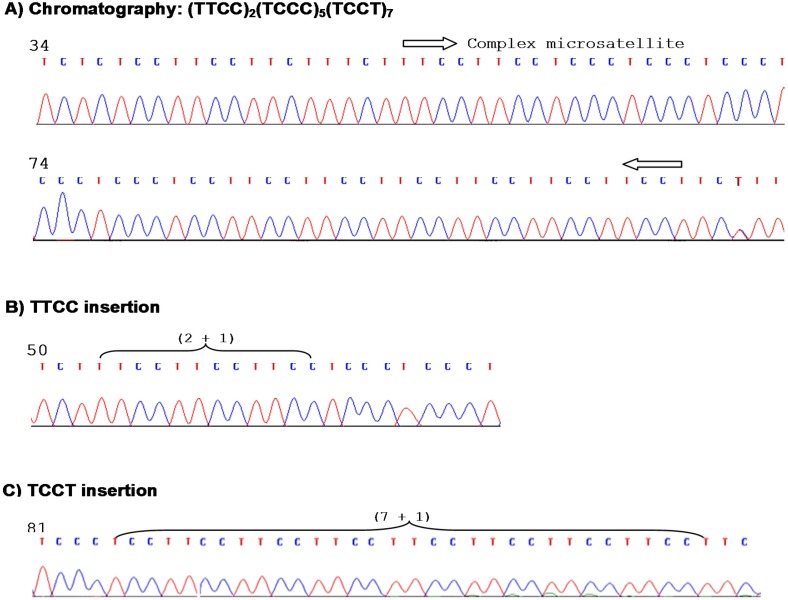
Chromatography of this microsatellite and representative polymorphisms **(A)** Sequencing chromatography of this microsatellite without polymorphisms, showing quality of the DNA sequencing data. **(B)** A sequence chromatography showing a single copy TTCC insertion. **(C)** A sequence chromatography showing a single copy TCCT insertion.

**Figure 3:**
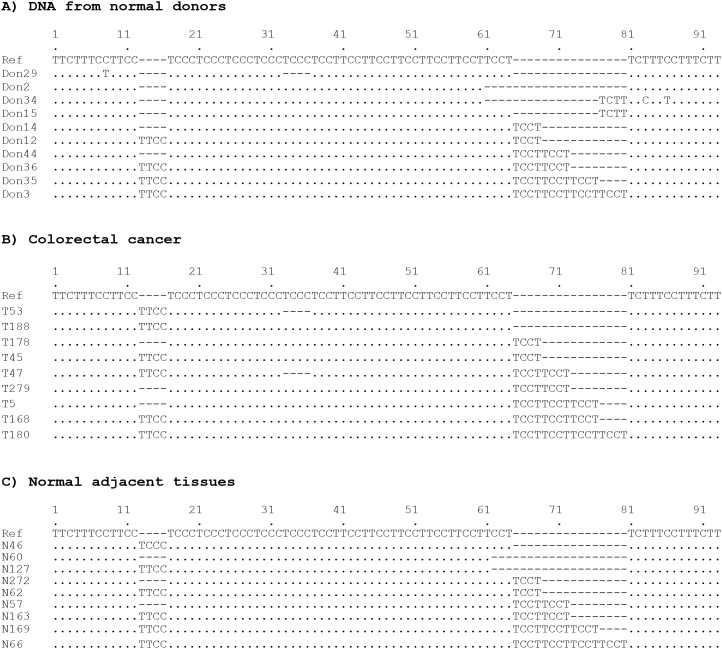
Polymorphic types of the complex microsatellite Presented sequences represent different types of polymorphic alterations observed in healthy donors **(A)**, colorectal cancer **(B)** and normal adjacent tissues **(C)**.

To characterize this repetitive sequence, we evaluated its polymorphisms and possible clinical implications in colorectal cancer. We first assessed its polymorphic changes in healthy subjects using the DNA sequence of NC_000007.14 as a reference sequence. Genomic DNA was amplified by PCR for conventional sequencing analysis. Figure 2 shows the chromatography of the sequencing data and representatives of polymorphic changes. Our results showed that this CT-enriched repetitive sequence is instable with polymorphic variations in 82 of 150 (54.7%) healthy donor subjects when compared to the reference sequence. We further analyzed the variant patterns and location in this site. The results showed that polymorphic variations mainly occurred in the tetranucleotide tandem repeats (TCCT)_7_ and (TTCC)_2_. The former accounted for 73.8% of overall polymorphic changes in this complex microsatellite and the latter denoted 24.3% of variations. The polymorphisms in the (TCCC)_5_ site were rare accounting only for 1.9% of overall polymorphisms (Table 1). As for the polymorphic patterns, the (TTCC)_2_ site was featured with insertions of a single copy TTCC, but the (TCCT)_7_ exhibited insertions of 1 - 4 copies of TCCT as main polymorphic forms. A single copy deletion of TCCT was detected in 14.6% of cases. A rare variant of a single copy TCTT insertion was noted in 2.9% of cases (Table 1). Figure 2B & 2C demonstrates the representative chromatography of single copy TTCC and TCCT insertions in healthy donors. Figure 3A shows the representative types of polymorphisms in healthy donors.

**Table 1:** Microsatellite polymorphisms compared to reference sequence

	Donors	NAT	CRC
	(*n* = 150)	(*n* = 143)	(*n* = 158)
**Polymorphic frequency**			
Polymorphism (%) ^1^	82 (54.7)	75 (52.4)	122 (77.2)
Non-polymorphism (%)	68 (45.3)	68 (47.6)	36 (22.8)
**Types and rates**			
TTCC site			
TTCC insertions (%)^2^	25 (24.3)	23 (23.5)	36 (20.9)
TCCC site			
TCCC deletions (%)	2 (1.9)	1 (1.0)	8 (4.7)
TCCC insertion (%)	0	1 (1.0)	5 (2.9)
Subtotal (%)	2 (1.9)	2 (2.0)	13 (7.6)
TCCT site			
TCCT deletions (%)	15 (14.6)	12 (12.2)	11 (6.4)
TCCT insertions (%)			
7 + 1 (%)	15 (14.6)	14 (14.3)	34 (19.8)
7 + 2 (%)	31 (30.1)	25 (25.5)	48 (27.9)
7 + 3 (%)	8 (7.8)	11 (11.2)	22 (12.8)
7 + 4 (%)	4 (3.9)	9 (9.2)	5 (2.9)
TCTT insertions (%)	3 (2.9)	2 (2.0)	3 (1.7)
Subtotal (%)^3^	76 (73.8)	73 (74.5)	123 (71.5)
**Total (PF/V)**^4^	**103 (1.26)**	**98 (1.31)**	**172 (1.41)**

CRC, colorectal cancer; NAT, normal adjacent tissues

^1^ Subjects (percentage) with polymorphisms compared to reference sequence. p<0.01, compared to NAT and donors.

^2^ p<0.05, compared to TCCC site. % in parenthesis indicates percentage in overall variations.

^3^ p<0.01, compared to TTCC and TCCC sites.

^4^ PF/V, polymorphic frequency per site; more than one type of polymorphisms in some sites.

We further analyzed the polymorphisms of this microsatellite in 158 colorectal cancer and 143 normal adjacent colorectal tissues (NAT). Results showed that in the NAT samples, polymorphic frequency, types and location in this complex microsatellite were pretty similar to those in the healthy donors. In the colorectal cancer tissues, the variant types and spectra were also similar to those in NAT and healthy donors, but when compared to reference sequence, the polymorphic frequency was higher at 77.2% in tumors vs. 52.4% in NAT (p<0.01). Figure 3B & 3C shows the representative types of polymorphisms in colorectal cancer and normal adjacent tissues. The polymorphic types and rates of this complex microsatellite in colorectal cancer and normal adjacent tissues are summarized in Table 1. These data suggest the instability of this complex microsatellite in carcinogenic progression.

**Figure 4:**
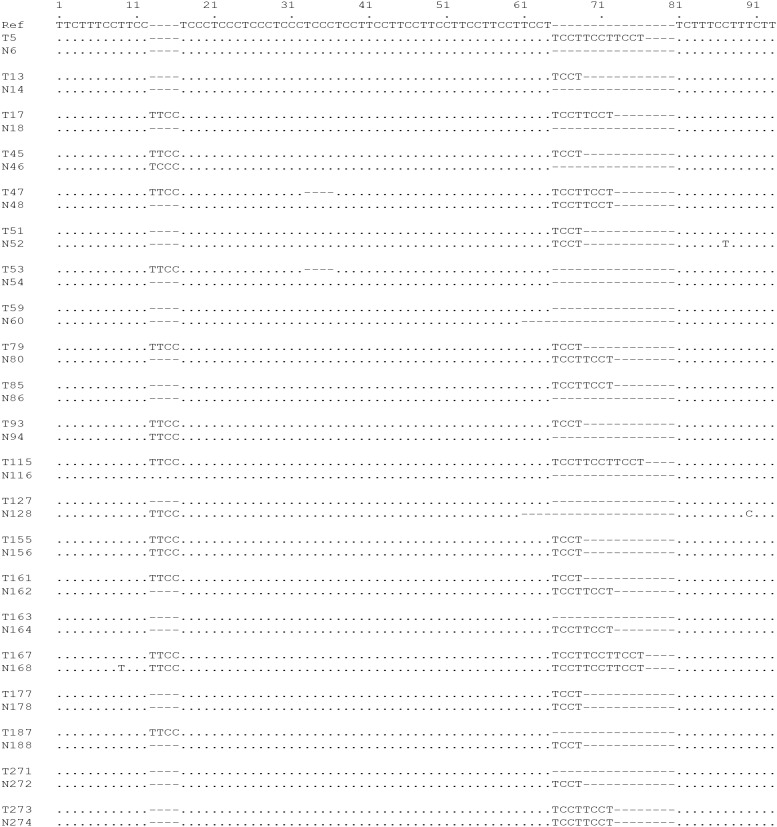
Polymorphic variations in sporadic colorectal cancer DNA sequences of this complex microsatellite from paired colorectal cancer and normal adjacent specimens were subjected to paired polymorphic analyses. Data show that expansions, contractions or both that occurred in some tumors during tumorigenesis.

To further address this question, we performed a pair-wise analysis of the polymorphic alterations in this microsatellite between the tumor tissues and matched NAT. In this study, a total of 121 pairs of colorectal specimens were collected and analyzed, and compared to the NAT, 88 (72.7%) of tumors displayed expansions, contractions or both in this complex microsatellite, suggesting that rapid cell proliferation and DNA replication during cancer development may stimulate polymorphic alterations. Figure 4 shows representative polymorphic variations in tumors in comparison with NAT samples.

### Negative association of the complex microsatellite polymorphisms with lymphatic metastasis of colorectal cancer

In view of the high polymorphic variations, we further assessed the clinical significance of this complex microsatellite. A cross-analysis of the polymorphisms with clinicopathological parameters collected from 158 colorectal cancer was performed. As demonstrated in Table 2, tumors with polymorphic alterations had less lymph node metastasis (p<0.001) compared to those with no polymorphic changes, suggesting its potential as a prognostic marker. Polymorphisms of this microsatellite had no correlation with patient age, sex or tumor size.

**Table 2:** Correlation between microsatellite polymorphisms and clinicopathological parameters in colorectal cancer

	Colorectal Cancer (*n* = 158)		
	Polymorphisms	Non-polymorphisms	P-Value
Subtotal	122 (77.2)	36 (22.8)	
Age (yr)			0.8508
Mean	60.8	61.7	
Median	65.0	63.0	
Range	23.0-89.0	45.0-84.0	
Sex			0.3653
Male	65	16	
Female	57	20	
Tumor size (cm)			0.8008
Mean	5.7	6.1	
Median	4.2	4.5	
Range	2.3-11.8	3.5-10.3	
Lymph node metastasis			0.0001
Positive	35 (28.7%)	27 (75.0%)	
Negative	87 (71.3%)	9 (25.0%)	

### Better disease-free survival of patients with polymorphisms of this complex microsatellite

**Figure 5:**
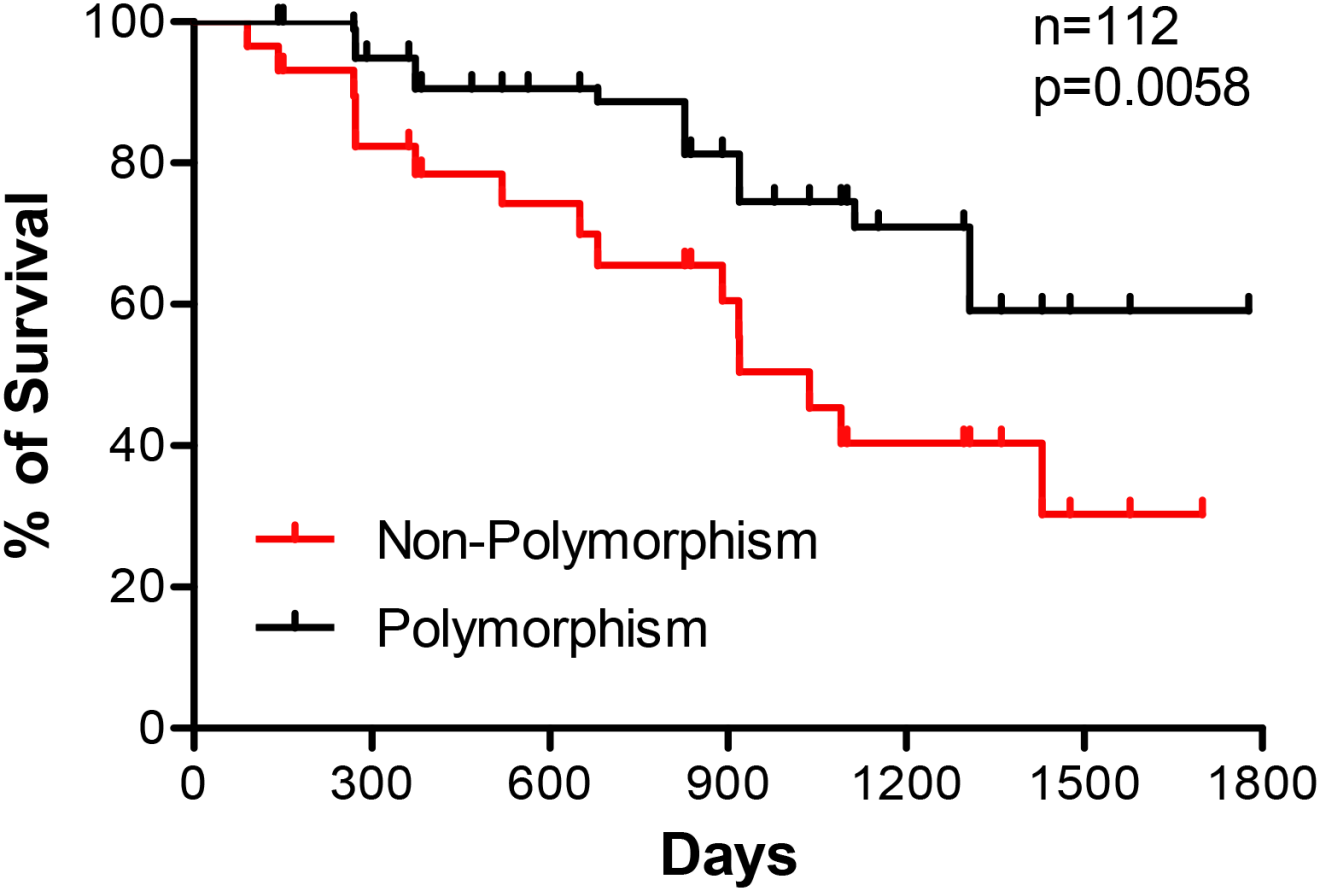
Correlation of microsatellite polymorphisms with disease-free survival of colorectal cancer patients Kaplan-Meier plots of patient survival were used for survival analysis. Data show a better survival of patients with polymorphic variations compared to those with no changes (n=112, p=0.0058).

With the finding of negative indication in lymph node metastasis, we further observed the correlation of the microsatellite instability with patient survival. We followed up the colorectal cancer patients subjected to the polymorphic analysis of this complex microsatellite, and survival data were obtained in 112 patients. Kaplan-Meier plots showed that patients with polymorphic variations of this complex microsatellite had significantly better disease-free survival (n=112, p=0.0058) (Figure 5). This data suggests that instability of this complex microsatellite may be a prognostic marker of colorectal cancer.

## DISCUSSION

Colorectal cancer is a serious public health issue. Through decades of efforts, colorectal cancer biomarkers, including microsatellites, have contributed to the diagnosis and prognosis, as well as prediction of treatment response. In this study, we identified and characterized a novel microsatellite marker that is located at Chromosome 7q33.

This microsatellite is composed of three tetranucleotide tandem repeats, (TTCC)_2_(TCCC)_5_(TCCT)_7_. Of the three different repeats, the (TCCT)_7_ site was most instable and accounted for majority of the polymorphic variations, followed by the (TTCC)_2_ while the (TCCC)_5_ site is virtually stable with very rare variations. Polymorphisms of microsatellites in tumors are often featured with expansions (insertions) and/or contractions (deletions) (27). Similarly, polymorphic type analyses showed that expansions or contractions were detected and that in some cases, expansions and contractions both occurred. However, frequency of the expansions or contractions varied. In the (TCCT)_7_ site, TCCT insertions, varied from one to four copies, were the main polymorphic forms and deletions with one copy of TCCT were a less event. Polymorphisms at the (TTCC)_2_ site were less frequent and featured with insertions of a single copy of TTCC. Mutations and/or epi-mutations in genes of DNA mismatch repair system (MMR), such as MLH1, MSH2, MSH6 and PMS2, leads to alterations in repeated DNA sequences (i.e., microsatellites), so-called microsatellite instability (MSI) [33]. However, why these tetranucleotide repeats demonstrated differential frequency of polymorphisms is currently unclear.

Comparative analyses between healthy donors, colorectal cancers and adjacent normal tissues demonstrated that polymorphisms occurred in all three cohorts of subjects when compared to the reference sequence. In these specimens, the polymorphic types were similar, but the polymorphic frequency was significantly higher in the tumor tissues than in NAT and healthy donors (p<0.01), indicating microsatellite instability during the tumorigenesis. This was further confirmed by paired analyses between colorectal cancers and matched normal adjacent tissues. Data showed that 72.7% of tumors displayed insertions, deletions or both in this complex microsatellite when compared to matching normal adjacent tissues. These data suggests that rapid cell proliferation and DNA replication during cancer development may push polymorphic alterations of this microsatellite.

Classic microsatellite markers used clinically are monocleotide and dinucleotide repeats, and their MSI occurs in approximately 15% of sporadic colorectal cancer [33]. However, a specific type of MSI, named elevated microsatellite alterations at selected tetranucleotide repeats (EMAST), may occur in up to 60% of sporadic colorectal cancer [34]. This complex microsatellite identified here is composed of three tetranucleotide repeats, (TTCC)_2_(TCCC)_5_(TCCT)_7_, which may explain the high polymorphic frequency of 72.7% in total of three sites in tumors when compared to normal adjacent tissues. This complex microsatellite may be a new complex EMAST.

This study also revealed that the polymorphisms of this complex microsatellite associated negatively with local lymph node metastasis, a critical factor for patient prognosis. This observation is consistent with literature reports that colorectal tumors with high MSI levels had less metastasis and better prognosis. Thus the patients with high MSI tumors have a higher disease-free survival rate compared to those without MSI [29, 35–37]. Indeed, a survival analysis in this study exhibited that the patients with polymorphic variations in this microsatellite had better disease-free survival, suggesting that this complex microsatellite may be a new positive prognostic marker of colorectal cancer. We have not a clear answer yet for decreased metastasis of the tumors with MSI, but the favorable prognosis may be ascribed to the better response to DNA damage agent 5-fluorouracil, a first-line drug for colorectal cancer.

Stool DNA has become a useful diagnostic specimen for disease screening and diagnosis. For instance, stool DNA kit (PreGen-Plus, MA) is developed to detect a panel of DNA markers involved in the colorectal cancer [38, 39]. It may be of potential value of early diagnosis or prognosis prediction by using the developed stool DNA techniques to exam the polymorphic changes in this complex microsatellite. The presence of a new polymorphism in this microsatellite in the stool DNA may indicate the presence of a tumor mass or a better prognosis for patients with developed colorectal cancer.

In summary, we identified and characterized a complex microsatellite that is located at Chromosome 7q33. This microsatellite has two polymorphic hot spots, (TCCT)_7_ and (TTCC)_2_, and polymorphic alterations of these spots positively associated with survival of the colorectal cancer, being a potential prognostic marker of this disease. A large cohort of prospective study may be merited to define the prognostic value of this microsatellite in colorectal cancer and the specificity and sensitivity of this complex microsatellite as a prognostic marker.

## MATERIALS AND METHODS

### Ethics statement

IRB protocols were approved by Hunan Cancer Hospital Committee for Research Involving Human subjects.

### Human sample procurement

With approved IRB protocols, we procured a total of 158 sporadic colorectal cancer and 143 normal adjacent colon specimens in this study, which were surgically resected from Hunan Cancer Hospital. All specimens were quality-controlled by pathologists. Clinicopathological data, including patient age and sex, and tumor size, differentiation, and lymph node metastasis were collected. Normal adult genomic DNA was extracted from blood of healthy donors. Registered patients were followed up for 5 years of disease-free survival.

### DNA extraction

Genomic DNA from surgical specimens was extracted as previously described [30]. After being completely dissolved in TE buffer (10 mM Tris.Cl and 1 mM EDTA, pH 8.0), DNA was quantitated at 260 nm using a spectrophotometer (Beckman, CA).

### PCR and DNA sequencing

Genomic DNA (200 ng) were used for PCR amplification at 95°C for 45 sec, 60°C for 30 sec, and 72°C for 90 sec for 30 cycles, after an initial denature at 95°C for 5 min. Forward primer was 5’GAA GTA TAA GAT TTT TCA CTC ATA G, and reverse primer was 5’GAA AGG AGA ATC ACT TGA ACC T GGG. PCR products were purified with PCR DNA purification kit (Qiagen, CA) and the sequences were read with a nested primer (5’CAT GCC AAT TGC CTT CTA TG) by a commercial company.

### Alignment of DNA sequences and data analysis

Sequence chromatograms were visualized and edited using Sequencher v4.6 (Gene Codes Corporation, MI) (24). Edited DNA sequences were saved as text files and contigs containing all sequences for a particular group. Pair-wise comparisons were assembled and exported as a NEXUS file. The 5' end of the microsatellite was marked by a TTCT motif for alignment. Gaps were inserted to indicate the insertions or deletions. The resulting alignments were edited using MacClade v4.06 (Sinauer Associates, Inc., MA) and analyzed for polymorphic changes. The microsatellite and flanking sequences in the database of the National Center for Biotechnology Information were used as a reference.

### Statistical analysis

Chi-square tests of independence, or Fisher’s exact tests when appropriate, were used to examine the relationship of microsatellite polymorphisms with tissue type, as well as with the clinical parameters of the cancer, such as gender, tumor differentiation, and lymph node metastasis. Analysis of variance was used to compare the polymorphism rates between sites. Independent group *t*-tests were used to assess the relationship of polymorphisms with patient age and tumor size. Patient survival was plotted by Kaplan-Meier analysis. Results were considered statistically significant with p<0.05. All analyses were performed using SAS v9.1 software, (SAS Institute Inc., NC).
